# A retrospective analysis of iv ketamine outcome on hospitalisations in an unselected psychiatric sample

**DOI:** 10.1017/neu.2024.18

**Published:** 2024-04-25

**Authors:** Karl Sandström, Olli Kampman, Peter Asellus

**Affiliations:** 1 Department of Clinical Sciences (Psychiatry), Umeå University, Umeå, Sweden; 2 Faculty of Medicine, Department of Clinical Medicine (Psychiatry), University of Turku, Turku, Finland; 3 Department of Psychiatry, The Wellbeing Services County of Ostrobothnia, Finland; 4 Faculty of Medicine and Health Technology, Tampere University, Tampere, Finland; 5 Department of Psychiatry, The Pirkanmaa Wellbeing Services County, Tampere, Finland

**Keywords:** Ketamine, mood disorders, depressive disorder, treatment-resistant, antidepressive agents

## Abstract

**Objective:**

This study aims to explore the outcome with iv ketamine treatment in a real-world clinical setting, primarily measured as posttreatment days hospitalised.

**Methods:**

The psychiatric medical records of 46 patients having received iv ketamine on a psychiatric treatment indication between 2015 and 2018 were retrospectively examined. Analysis comparing the number and duration of hospital admissions before and after ketamine treatment as well as logistic regression analysis to investigate clinical predictors of effectiveness, were performed. To assess patients’ severity of depressed symptoms records were screened for MADRS-S scores.

**Results:**

No significant difference between pre- and posttreatment hospital days (*p* = 0.170), or number of hospitalisations (*p* = 0.740) were found. The response rate was 31% and remission rate 21%. None of the predictors showed statistical significance in the logistic model.

**Conclusion:**

Iv ketamine treatment showed effectiveness in reducing depressive symptoms even with complex patients in a real-world clinical setting. However, this did not translate to a reduction in hospitalisation. Highlighting the multifaceted challenges posed when implementing iv ketamine treatment in clinical practice.

## Significant outcomes

- Iv ketamine produced a significant reduction in depressive symptoms even when administrated to a heterogenous population under real-world clinical conditions. The results support the view of iv ketamine as an efficacious treatment in a variety of settings.
- The reduction in depressive symptoms did not translate to a significant reduction in the need for inpatient care. This is in contrast with an earlier similarly designed study and indicate factors other than the antidepressant effect of ketamine being significant in the need for hospitalisation in corresponding populations.
- The study highlights both treatment specific and extra-pharmacological factors as clinical challenges when implementing iv ketamine treatment in clinical practice and further emphasise the importance of structured treatment and evaluation protocols.

## Limitations

- The study sample was small and heterogeneous, did not include a control group and the patients were recruited from a single study site.
- The documentation in the medical records was occasionally incomplete or unclear and both the diagnostic and treatment protocol lacked stringency.
- The rationale for choosing iv ketamine instead of other treatments, and the occurrence of side effects, is unknown. The open-label nature of the study includes the possibility for a significant placebo effect.

## Introduction

Ketamine was first developed in the 1960s as a rapidly acting anaesthetic (Corssen and Domino, 1966). It is a racemic mixture of its two enantiomers, (R)- and (S)-ketamine and derived from phencyclidine (PCP) (Zanos *et al*., 2018). Ketamine is classified as a noncompetitive NMDA-receptor antagonist. However, it exhibits a complex pharmacological profile with interactions at a range of receptors (Zanos *et al*., 2018). Ketamine has since its initial introduction gained prominence as an antidepressant drug (Corriger and Pickering, 2019). This was largely initiated by the publication of the first placebo-controlled study following a growing body of preclinical research (Berman *et al*., 2000). Subsequently, the promising initial results has prompted further investigations, in large part due to the ability of sub-anaesthetic doses of intravenous (iv) ketamine to produce a rapid and robust antidepressant effect in both unipolar and bipolar depression. (Wan *et al*., 2015; Corriger and Pickering, 2019; Jelen and Stone, 2021). Recently, the effectiveness of ketamine has encouraged research into its usefulness as an alternative to electroconvulsive therapy (ECT) (Ekstrand *et al*., 2022; Anand *et al*., 2023). However, the effectiveness of ketamine treatment on hospitalisation rates in a naturalistic setting remains understudied. With increasing clinical adoption of ketamine in the treatment of depression the need for investigation in a real-world clinical setting increase.

This study aims to explore the outcome with iv ketamine treatment in a real-world clinical setting, primarily measured as days spent as an inpatient.

## Methods

### Setting

The use of ketamine to treat psychiatric illness was not approved by the Swedish Medical Products Agency and there were no national or clinic-specific treatment guidelines during the period covered in this study. A survey conducted in 2016 by the Swedish Agency for Health Technology Assessment and Assessment of Social Services estimated that in Sweden in 2015 fewer than 75 patients received iv ketamine to treat depression ((SBU) 2017). The survey also reported that iv ketamine was viewed as an alternative to ECT and mainly offered to patients with treatment-resistant depression (TRD) and chronic suicidal ideation. TRD was in this instance defined as inadequate response to a minimum of two adequate antidepressants treatments. This is in line with the definition adopted by The US Food and Drug Administration (FDA) and the European Medicines Agency (EMA), that is, inadequate response to a minimum of two antidepressants despite adequacy of the treatment trial and adherence to treatment (McIntyre *et al*., 2023). However, a consensus definition of TRD does not currently exist (McIntyre *et al*., 2023).

The clinical setting was a University Hospital psychiatric clinic in the city of Umeå in northern Sweden. The clinic manages both inpatient and outpatient specialised care. Iv ketamine had been routinely used at the clinic during the years leading up to 2018 after which the treatment with iv ketamine was terminated by administrative decision. The clinic had no established dosing protocol for iv ketamine.

### Study design

This retrospective register based observational study was conducted to assess the outcome of iv ketamine on the need for inpatient care in a real-world population. All participants enrolled in the study were at least 18 years of age and were included based on having received iv ketamine on a psychiatric treatment indication between 2015 and 2018. As there were no national or local clinical guidelines for ketamine-treatment at the time, treatment was initiated based on clinical assessment at the discretion of the treatment-responsible doctor and off-label. No clinic-specific inclusion criteria were present. Study participants were screened and selected according to anaesthesia records by matching psychiatric treatments and anaesthesia procedures at the hospital care register between the years 2015 and 2018 since ketamine treatment was administrated via the anaesthesia department. In total, 46 patients were found eligible for inclusion. No exclusion criteria were used. The participants psychiatric medical records were then examined. Ethical approval for this study was provided by The Swedish Ethical Review Authority.

### Outcome measures

Two primary outcomes were chosen. First, the total number of days hospitalised, before and after treatment with iv ketamine, was measured. Second, the number of hospitalisations, before and after ketamine treatment, was measured. These measures were chosen because they reflected the study participants overall functioning and severity of illness. Our expectation was that iv ketamine would have been reserved for a patient population characterised by treatment resistance and chronicity, and that iv ketamine would be used as an alternative treatment to ECT. To investigate the impact of previous hospitalisation on our primary outcomes analysis was performed both on the total sample as well as the sub-group including only patients with at least one day’s pretreatment hospitalisation.

Based on the participants medical records, a starting point of iv ketamine treatment was defined. The starting point was set as the first day of the first index series of iv ketamine. From this starting date, a timespan of 365 days pre- and post-treatment was identified. Thereby, a consistent number of days pre- and post-ketamine treatment was obtained for each patient. This timespan was then examined regarding our primary outcomes for each patient.

To describe the study participants severity of depressed symptoms, self-reported Montgomery-Åsberg Depression Rating Scale score (MADRS-S; (Svanborg and Åsberg, 1994; Svanborg and Åsberg, 2001)) was registered when obtained, before and after the first index series with ketamine. In patients with no recorded MADRS-S scores (10 at baseline, 19 at endpoint) this data was replaced with a retrospectively estimated MADRS-S score through a review of the medical records by the clinical investigator (KS). The MADRS-S scale has been shown to have high concordance with the original MADRS scale (Mattila-Evenden *et al*., 1996; Svanborg and Ekselius, 2003). The clinical diagnosis used as an indication for ketamine treatment, sex, age and psychiatric comorbidity were registered. The total number of index series, as well as the number of individual ketamine treatments given, were also recorded. Response was determined as a symptom reduction greater or equal to 50% by the index-series of ketamine as measured by MADRS-S score (Nierenberg and DeCecco, 2001) and remission as a MADRS-S score less than 13 points (Svanborg and Ekselius, 2003).

As the medical records did not give a complete and satisfactory registration of the given treatment doses of ketamine for every individual treatment a mean dose for the sample could not be calculated. Therefore, a typical treatment dose was estimated from a random sample of 20 participants.

### Data analysis

Normality of the continuous variables (age, MADRS-S score, the number of hospitalisations and hospital days) were analysed with QQ plots. Wilcoxon signed rank test was used to compare pre- and posttreatment hospital days and hospital admissions. Paired samples *t*-test was used to compare pre- and posttreatment MADRS-S scores. Concordance between self-reported and estimated MADRS-S scores were analysed with independent samples *t*-test.

To explore associations for multicollinearity (*r* > 0,8) between background (sex and age) and clinical variables (diagnosis of mood disorder, number of ketamine treatments and -series, pre- and posttreatment hospitalisations, hospital days and MADRS-S scores) Spearman’s correlation coefficients were calculated. A logistic regression analysis with the presence of posttreatment hospital days as the dependent variable was performed including examining collinearity.

Statistical analysis was performed using IBM SPSS Statistics, version 25 (Armonk NY). The level of statistical significance was set at *p* < 0.05.

## Results

Baseline characteristics of the sample are presented in Table 1. Forty-six patients were identified as having received iv ketamine treatment at the clinic between 2015 and 2018. Of these, 37 had complete, retrievable medical records, and 9 patients were referred from a different region with only partial medical records available.

**Table 1.** Baseline characteristics

	Male	Female			*N*
Sex	14 (30%)	32 (70%)			46
	**Min**	**Max**	**Median (IQR)**	**Mean (SD)**	**N**
Age	29	89	52 (28)	54 (17.0)	46
Nr of hospitalisations pretreatment	0	8	1 (3)	1.6 (1.8)	37
Nr of hospitalisations posttreatment	0	24	1 (3)	2 (4.1)	37
Nr of inpatient days pretreatment	0	329	6 (35)	30 (59.8)	37
Nr of inpatient days posttreatment	0	365	8 (53)	43 (77.4)	37
MADRS-S baseline	15	54	32 (13)	32 (8.6)	45
MADRS-S endpoint	4	46	22 (18)	23 (10.9)	43
Nr of treatments received	4	127	10 (24)	22 (25.3)	46
Nr of treatments in index series	4	61	9 (15)	18 (17.7)	46
	**Yes (%)**	**No (%)**			**N**
ECT pretreatment	12 (31)	27 (69)			39
Suicide attempt adjacent to treatment start	5 (12)	37 (88)			42
Suicide attempt pretreatment	8 (21)	30 (79)			38
ECT posttreatment	5 (14)	32 (86)			37
Suicide attempt posttreatment	3 (9)	32 (91)			35
**Diagnosis**	**N** (%)				
Depressive disorder (F32x, F33x)	20 (43.5)				
Bipolar Disorder (F31x)	13 (28.3)				
Anxiety disorder (F4xx)	8 (17.4)				
Other diagnosis	5 (10.9)				

Pretreatment = 365 days prior to start of index series.

Posttreatment = 365 days after start of index series.

SD = Standard Deviation; IQR = Interquartile range.

A wide diagnostic spread was observed. Thirty-three patients (72%) had a mood disorder ICD-10 diagnosis, of which 20 (43%) had a diagnosis of depression and 13 (28%) a diagnosis of bipolar disorder. Of the latter, 46% had bipolar depression and 54% hade an unspecified bipolar disorder. Of those with anxiety disorders as a main diagnosis, 50% had a F41 diagnosis, 38% had a F43 diagnosis and 12% had a F45 diagnosis. Those with other diagnoses included 40% with an eating disorder, 40% with a neuropsychiatric disorder and 20% with a personality disorder as the main diagnosis. In total, twelve patients (26%) had registered psychiatric comorbidities, with personality disorders being the most common (9%).

Thirty-one per cent had received ECT pretreatment and 14% received ECT posttreatment during follow-up.

The estimated given mean ketamine dose per treatment was 45 mg (SD 12.7) and roughly in line with the widely adopted dose of 0,5 mg/kg (Correia-Melo *et al*., 2018). Noteworthy was the occasional inclusion of esketamine. When encountered, esketamine was converted to an equivalent dose of racemic ketamine using accepted ratios (Correia-Melo *et al*., 2018).

### Primary outcomes

Thirty-seven patients were eligible for the main analysis regarding our primary outcomes. There was no significant difference between pre- and posttreatment hospital days (*p* = 0.170, *t*-test), or number of hospitalisations (*p* = 0.740). Neither was there a difference between pre- and posttreatment hospital days (*p* = 0.230), or hospital admissions (*p* = 0.943) in the sub-group (*n* = 23) including only patients with at least one day’s pretreatment hospitalisation.

### MADRS score

Estimated MADRS-S scores exhibited equal variance and no significant difference to the self-reported scores (*p* = 0.657 pretreatment, *p* = 0.442 posttreatment, *t*-test). MADRS-S score was significantly reduced after the initial index-series of ketamine for both self-reported (*p* = 0.001, mean difference 9 points, SD 10.9, *t*-test) and combined, that is, self-reported and estimated scores (*p* < 0.001, mean difference 9 points, SD 11.3, *t*-test).

Thirteen patients (31%) achieved a response and nine (21%) remission.

### Post hoc analysis

The results of the Spearman correlation matrix are presented in Table 2. A moderate negative correlation between age and MADRS-S score pretreatment can be noted (*r* = −0.553, *p* < 0.000).

**Table 2.** Spearman’s correlation for MADRS-S pre- and posttreatment scores and background variables

		MADRS-S score pretreatment	MADRS-S score posttreatment
Sex	Correlation Coefficient	−0.091	0.152
	Sig.	0.553	0.330
	N	45	43
Age	Correlation Coefficient	−0.553	−0.092
	Sig.	0.000	0.557
	N	45	43
ICD diagnosis (F3xx, F4xx, other)	Correlation Coefficient	0.084	0.242
	Sig.	0.583	0.119
	N	45	43
Nr of treatments in index-series	Correlation Coefficient	0.130	−0.092
	Sig.	0.395	0.558
	N	45	43
MADRS-S score pretreatment	Correlation Coefficient	1.000	0.376
	Sig.	.	0.014
	N	45	42
MADRS-S score posttreatment	Correlation Coefficient	0.376	1.000
	Sig.	0.014	.
	N	42	43
Nr of days hospitalised pretreatment	Correlation Coefficient	0.209	−0.048
	Sig.	0.222	0.783
	N	36	35
Nr of days hospitalised posttreatment	Correlation Coefficient	0.340	0.202
	Sig.	0.043	0.245
	N	36	35
Nr of admissions pretreatment	Correlation Coefficient	0.226	−0.058
	Sig.	0.185	0.741
	N	36	35
Nr of admissions posttreatment	Correlation Coefficient	0.288	0.114
	Sig.	0.088	0.515
	N	36	35

Logistic regression, with the dependent variable as posttreatment days hospitalised was performed to investigate the impact of socio-demographic and clinical predictors on treatment outcome (Table 3). The independent variables in the model included sex, age, binary mood disorder diagnosis (F3 diagnosis yes/no), the number of received ketamine treatments and MADRS-S score change during index treatment. None of the predictors showed statistical significance in the model.

**Table 3.** Logistic regression with the presence of posttreatment hospital days as the dependent variable

	*p*-value	OR	95% CI for OR	
			Lower	Upper
**Categorical variables**				
(Intercept)	0.042	1.875		
Sex	0.369	0.976	0.936	1.018
F3xx diagnosis or other	0.730	1.282	0.132	5.265
**Continuous variables**				
(Intercept)	0.068	1.800		
Age	0.250	0.975	0.935	1.018
Nr of treatments in index-series	0.649	1.014	0.976	1.054
MADRS-S score change during treatment	0.621	1.015	0.956	1.078

## Discussion

Iv ketamine treatment produced a significant reduction of depressive symptoms. This is consistent with findings seen in other studies (Marcantoni *et al*., 2020; McIntyre *et al*., 2021) supporting the view of iv ketamine as an efficacious treatment in a variety of settings. Compared to a 2018 retrospective clinical study including TRD patients assessed as ultra-resistant (Thomas *et al*., 2018) and a 2020 meta-analysis examining the efficacy of ketamine on TRD (Marcantoni *et al*., 2020) remission rates were higher and response rates lower. Both the response and remission rates were higher in our sample compared to a Canadian, retrospective, naturalistic study examining the effectiveness of iv ketamine on depressive symptoms, suicidal ideation and functional disability in major depressive disorder and bipolar disorder (McIntyre *et al*., 2020). A 2024 retrospective study examining clinical outcomes of iv ketamine for depression found both lower response and remission rates after 6 weeks of treatment compared to our sample (Pfeiffer *et al*., 2024). However, in the KetECT and ELEKT-D studies, examining the comparative effectiveness of iv ketamine to ECT, remission rates in the iv ketamine sample was distinctly higher than seen in our study sample (Ekstrand *et al*., 2022; Anand *et al*., 2023). The same was found in a 2022 retrospective analysis of iv ketamine for depression in a real-world setting (McInnes *et al*., 2022). These inconsistencies might reflect the diagnostic heterogenicity and the severity of depressed mood in our study sample. It underlines the importance of maintaining a strict treatment protocol in the clinical setting to reliable produce expected results. Lack of a strict protocol increases the risk of uncertain treatment outcomes.

Regarding our primary endpoints it´s noteworthy that even though a significant reduction in symptom score and hence treatment effect was observed, this did not translate to a significant reduction in the need for inpatient care. This is in contrast with an earlier similarly designed study where treatment with oral ketamine significantly reduced the need for inpatient care for patients with TRD and post-traumatic stress disorder (Hartberg *et al*., 2018). The lack of significant effect regarding our primary endpoints may therefore be due to the presence of factors other than the pharmacological antidepressant effect of ketamine such as potential misuse, diagnostic variability and expectancy bias.

In the multivariate analysis, neither sociodemographic factors such as age and sex nor the treatment-specific variable of number ketamine treatments received were found to be significantly affecting the posttreatment days hospitalised. This is in line with previous investigations regarding clinical predictors for effectiveness of iv ketamine (Rong *et al*., 2018) and brings into focus the need to develop clinically useful markers to predict the effectiveness of ketamine treatment. Efforts investigating clinical variables, biomarkers and neuroimaging results are ongoing (Rong *et al*., 2018; Meshkat *et al*., 2023). However, further research is needed before these findings can be reflected in the development of feasible clinical tools to personalise ketamine treatment strategy.

Interestingly, nor had a mood disorder diagnosis or reduction of depressive symptoms an association on hospitalisation in the multivariate analysis. This indicates that extra-pharmacological factors, such as social isolation, might contribute to the need for inpatient care in the study sample. Previous research has shown patients with strong social support being less likely to be admitted to psychiatric hospital (Albert *et al*., 1998; Van Veen *et al*., 2019) and a 2022 meta-analysis examining the social dimension of suicidal aetiology emphasised social isolation as a risk factor for suicide (Motillon-Toudic *et al*., 2022).

Worth highlighting is the wide diagnostic spread seen in the study sample. It is possible that in some cases treatment with ketamine was initiated based on a clinical presentation of depressed mood, which was not reflected in an ICD-10 diagnosis encoded in the medical records. In this context it is to be considered that the study aimed to explore the outcome with iv ketamine in a real-world clinical setting, without strict inclusion criteria commonly seen in randomised trails and that the diagnoses were based on clinical evaluations and not on structured diagnostic interviews, which could have provided a higher diagnostic accuracy. Furthermore, the prevalence of comorbidity is low. Other studies have shown a high degree of comorbidities in TRD populations receiving iv ketamine (Thomas *et al*., 2018). In particular, the frequency of comorbid personality disorders is low in our study sample compared to what is to be expected (Tyrer *et al*., 2015). Taken together, this emphasises the importance of maintaining a meticulous diagnostic process in the clinical setting.

It has previously been noted that in a sample of TRD patients characterised by high chronicity, treatment resistance and chronic suicidal ideation, the established treatment regime with 0,5 mg/kg may not be sufficient to produce symptom improvement (Ionescu *et al*., 2019). This finding could be applicable to some patients who could have received lower doses during treatment. Unfortunately, due to lack of data we were not able to estimate the number of patients with suboptimal dosage. Furthermore, both iv racemic ketamine and iv esketamine were used in this study sample. A 2021 meta-analysis comparing iv racemic ketamine to intranasal esketamine indicate that racemic ketamine could be superior to esketamine for treating depression (Bahji *et al*., 2021). To our knowledge there is no evidence indicating that iv ketamine and iv esketamine can be used interchangeably when treating psychiatric illness. However, a 2016 multi-centre, randomised, placebo-controlled trial exploring the efficacy of iv esketamine on 30 patients with TRD observed an antidepressant effect (Singh *et al*., 2016). The interchangeable use of ketamine and esketamine might contribute to the observed lack of congruity regarding some of our findings compared to prior research.

The open-label nature of this kind of study comes inherently with biases. As is often the case with these kinds of clinical studies, the study sample was small and heterogeneous, did not include a control group and the patients were recruited from a single study site. These factors limit the generalisability of the study’s findings. Furthermore, it lacks the stringency in treatment protocol found in randomised clinical trials, evident in both the patient selection and treatment regime. The documentation in the medical records was occasionally incomplete or unclear. The study lacks specific data on adverse events and drop-out frequency, areas crucial to be able to correctly judge the risk–benefit profile of a given treatment. This is of particular importance in off-label treatment, especially when patient inclusion seemingly is expanded into diagnostic areas containing limited or no scientific evidence. The precise rationale for choosing iv ketamine over established treatments or whether patients were deemed resistant to ECT pretreatment, is not included in the study data. As ketamine was administered off-label without established treatment guidelines, no clear directives whether ECT was to be administered prior to ketamine were in place, further highlighting a problem with off-label use of ketamine in such a setting. Further management strategies post-treatment is not included in the study data.

As was noted previously, the comparative effectiveness of iv ketamine to ECT have been addressed in two previous studies (Ekstrand *et al*., 2022; Anand *et al*., 2023). The results indicate ECT being superior to iv ketamine in hospitalised patients. As ketamine was mainly regarded as an off-label option for TRD during the years included in this study, we expected to find a population characterised by treatment resistance and chronicity with a high degree of hospitalisation. Instead, a large variation in the study sample was found. Given the sample characteristics, a more precise description of the treatment related considerations when initiating iv ketamine at that time would have been an asset. The comparable effectiveness of iv ketamine to treatments such as second-generation antipsychotics and combined antidepressants in TRD has neither been established (McIntyre *et al*., 2021).

The open-label nature also suggests the possibility of a large placebo effect. A recent, triple-masked study, administrating sub-anaesthetic doses of iv ketamine to depressed patients undergoing anaesthesia for routine surgery showed no difference in antidepressant effect when comparing iv ketamine to saline-placebo (Lii *et al*., 2023). This suggests significant influence of expectancy bias and extra-pharmacological effects on treatment results. These findings might be relevant in explaining the results in this study showing a reduction in depressive symptoms but not in the need for inpatient care. However, a 2015 meta-analysis examining the effects of iv ketamine on major depressive disorder and bipolar disorder did not demonstrate a significant difference in effect size between open-label and participant-blind infusions (Coyle and Laws 2015). These inconsistencies further emphasise the need for additional research and clinical treatment guidelines.

The reinforcing and rewarding properties of ketamine together with its potential toxicity have brought concerns about potential misuse into focus (Liu *et al*., 2016). A 2022 review highlighted that the evidence is insufficient to dependably demonstrate the abuse liability of ketamine in depressed patients and that high quality RCTs have excluded patients with high abuse potential (Le *et al*., 2022). Case reports have observed ketamine dependance in depressed patients when not treated in a proper setting (Bonnet, 2015; Schak *et al*., 2016). It has previously been acknowledged that evidence is lacking regarding dose optimisation, treatment frequency and long-term efficacy of ketamine treatment in TRD patients (McIntyre *et al*., 2021). Altogether, this suggests a risk of treatment continuation based on factors other than treatment efficacy. These factors could have contributed to the administrative decision to terminate ketamine treatment at the clinic. Our study sample lack data on substance abuse, dissociation and euphoric effects during treatment and therefore cannot address this question further. To ensure that treatment continuation is based on increased patient functioning and to reduce potential abuse liability, treatment evaluation using objective measures, such as the need for hospitalisation, and not subjective experiences is prudent and a cautionary approach would be advisable.

The study highlights both treatment specific and extra-pharmacological factors as clinical challenges posed in a real-world study sample of patients found eligible for iv ketamine in psychiatric practice. Considering the multiple clinical questions waiting to be elucidated by further research the results in this study emphasise the importance of a determined treatment plan, governed by established guidelines and considering the diagnostic and social perspective as well as treatment specific factors. The study underlines the importance of maintaining a strict inclusion and treatment protocol with set evaluation criteria even in the clinical setting to facilitate the evaluation of treatment effects more precisely. Highlighting the difficulties associated with undefined inclusion and evaluation criteria in conjuncture with promising, but sometimes conflicting, scientific evidence. The main strength of this real-world study design is its potential for strong external validity in a clinical setting, which in part is due to the absence of exclusion criteria. However, the study results also highlight this possible strength as a limitation. Since initiating an off-label treatment without strict inclusion criteria might lead to suboptimal outcomes, as indicated by the lack of significant effect on our primary outcomes.

We believe that our primary outcome measures as such are suitable in conjunction with commonly used symptom rating scales in evaluation of treatment response in corresponding patient populations. Especially given the many clinical questions awaiting clarification, suggesting a cautionary approach. Otherwise, there is a risk of poorly focused treatments which might not provide the greatest benefit for the individual patient nor a best possible risk–benefit proposition.

## Supporting information

Sandström et al. supplementary material Sandström et al. supplementary material

## Supplementary material

The supplementary material for this article can be found at https://doi.org/10.1017/neu.2024.18.
