# Supplementary material for: A retrospective analysis of iv ketamine outcome on hospitalisations in an unselected psychiatric sample
Source: Acta Neuropsychiatr. 2024 Apr 25;37:e13. doi: 10.1017/neu.2024.18 (PMC13130326; doi:10.1017/neu.2024.18)
Supplement: Sandström et al. supplementary material [file S0924270824000188sup001.docx]

**Abstract**

Objective: This study aims to explore the outcome with iv ketamine treatment in a real-world clinical setting, primarily measured as posttreatment days hospitalized.

Methods: The psychiatric medical records of 46 patients having received iv ketamine on a psychiatric treatment indication between 2015-2018 were retrospectively examined. Analysis comparing the number and duration of hospital admissions before and after ketamine treatment as well as logistic regression analysis to investigate clinical predictors of effectiveness, were performed. To assess patients’ severity of depressed symptoms records were screened for MADRS-S scores.

Results: No significant difference between pre- and posttreatment hospital days (*p*=0.170), or number of hospitalizations (*p*=0.740) were found. The response rate was 31% and remission rate 21%. None of the predictors showed statistical significance in the logistic model.

Conclusion: Iv ketamine treatment showed effectiveness in reducing depressive symptoms even with complex patients in a real-world clinical setting. However, this did not translate to a reduction in hospitalization. Highlighting the multifaceted challenges posed when implementing iv ketamine treatment in clinical practice.

**Bullet points:**

Iv ketamine produced a significant reduction in depressive symptoms even when administrated to a heterogenous population under real-world clinical conditions. The results support the view of iv ketamine as an efficacious treatment in a variety of settings.

The reduction in depressive symptoms did not translate to a significant reduction in the need for inpatient care. This is in contrast with an earlier similarly designed study and indicate factors other than the antidepressant effect of ketamine being significant in the need for hospitalization in corresponding populations.

The study highlights both treatment specific and extra-pharmacological factors as clinical challenges when implementing iv ketamine treatment in clinical practice, and further emphasize the importance of structured treatment and evaluation protocols.
